# P-2082. Shifting Susceptibility: MIC Assessment in RPMI versus MHB for Daptomycin Non-Susceptible MRSA Isolates

**DOI:** 10.1093/ofid/ofae631.2238

**Published:** 2025-01-29

**Authors:** Callan Bleick, Samantha Vader, Aminah Lawal, Shaylyn Avery, Michael J Rybak

**Affiliations:** Anti-Infective Research Laboratory, Eugene Applebaum College of Pharmacy and Health Sciences, Wayne State University - Detroit (United States); Wayne State University School of Medicine, Department of Microbiology and Immunology - Detroit (United States), Detroit, Michigan; nti-Infective Research Laboratory, Eugene Applebaum College of Pharmacy and Health Sciences, Wayne State University - Detroit (United States), Detroit, Michigan; Anti-Infective Research Laboratory, Eugene Applebaum College of Pharmacy and Health Sciences, Wayne State University - Detroit (United States), Detroit, Michigan; 1. Anti-Infective Research Laboratory, College of Pharmacy and Health Sciences, Wayne State University, Detroit, MI, Detroit, Michigan; Eugene Applebaum College of Pharmacy and Health Sciences, Detroit, Michigan

## Abstract

**Background:**

Traditional minimum inhibitory concentration (MIC) screening, widely adopted in clinical microbiology labs, utilize Mueller-Hinton broth (MHB) to determine susceptibility breakpoints for bacterial organisms. The Roswell Park Memorial Institute (RPMI) medium supplemented with 5% Luria-Bertani Broth (LB) is commonly used in cell culture research to maintain cell health and improve viability. Additionally, it has been demonstrated to better mimic physiological conditions in humans. This study seeks to elucidate differences in MICs in 25 daptomycin non-susceptible (DNS) methicillin-resistant *Staphylococcus aureus* (MRSA) isolates.

**Methods:**

Twenty-five DNS-MRSA bacterial strains were utilized. Variations in daptomycin MICs between RPMI and MHB were determined via triplicate microbroth dilution (MBD). After 24 hours, 50 µL of resazurin was added to each 96-well plate, and MICs recorded. Both RPMI and MHB were supplemented with 50 mg/L of calcium, and pH levels verified (pH 7.30-7.50). We assessed differences in MICs between RPMI and MHB using the Mann-Whitney U test (*P* < 0.05).

**Results:**

The comparison of daptomycin MICs between RPMI and MHB revealed significant differences (*P* < 0.05) for the 25 DNS-MRSA bacterial strains examined. Specifically, in RPMI, MIC values ranged from 0.0125 to 2, whereas in MHB, MIC values ranged from 2 to 4. This suggests a notable impact of culture medium on the effectiveness of daptomycin against the tested bacterial strains. The study's findings underscore the importance of considering culture conditions when evaluating antimicrobial susceptibility, particularly for DNS-MRSA strains.

**Conclusion:**

Our comparative assessment of MICs using RPMI versus MHB revealed statistically significant differences in antimicrobial activity. These differences warrant further investigation across additional strains and antibiotics. Notably, while the breakpoint for daptomycin is 1 mg/liter for *S. aureus*, our MICs done in RPMI indicated susceptibility of previously recorded DNS strains with a MIC < 1 mg/L according to CLSI guidelines. These findings highlight the importance of considering potential shifts in MICs based on testing media differences and suggest a need for reevaluation of traditional susceptibility testing methods.

**Disclosures:**

Michael J. Rybak, PharmD, PhD, MPH, Abbvie, Melinta, Sionogi, Merck, T2Biosystems: Advisor/Consultant|Abbvie, Melinta, Sionogi, Merck, T2Biosystems: Grant/Research Support|Abbvie, Melinta, Sionogi, Merck, T2Biosystems: Speaker

